# Paolo Guidetti

**Published:** 2009-02-16

**Authors:** Robert Schwarcz

**Affiliations:** University of Maryland School of Medicine, Baltimore, MD (U.S.A)

**Figure:**
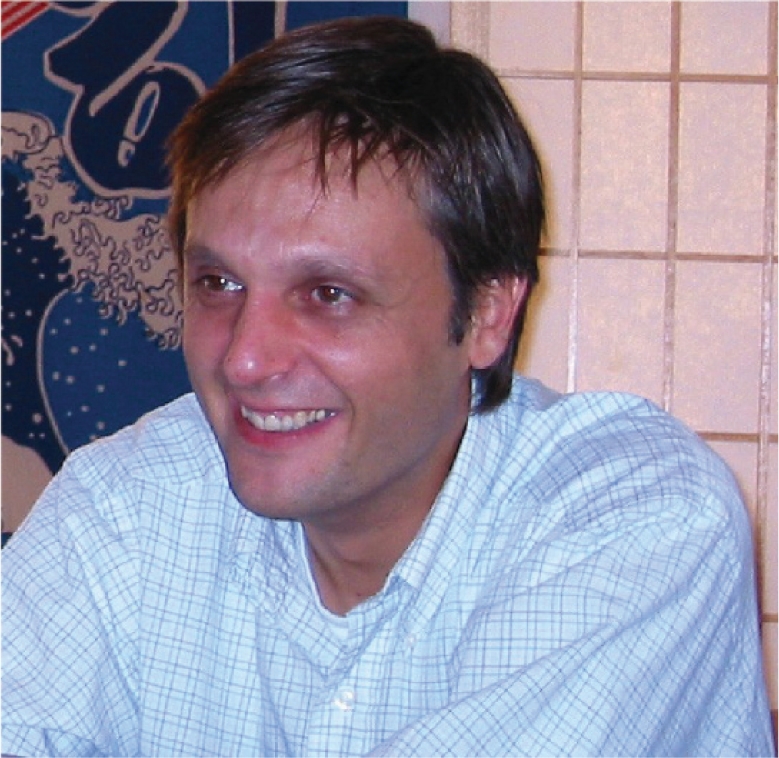
Paolo Guidetti

Paolo Guidetti passed away on December 28, 2007 at age 41, a victim of a brief, devastating bout with lymphoma. His death deprives the field of tryptophan research of one of its brightest and most promising young stars. We also mourn the loss of a unique individual, who not only had an outstanding mind but possessed an exceptional ability to inspire and to foster collaborations and friendships.

Paolo was born into a prominent family in Reggio Emilia, one of the major cities of Italy’s Emilia-Romagna region. During his graduate education at the nearby University of Modena, where he was mentored by the eminent neuroscientist Mario Baraldi, he began to develop a deep-seated interest in the brain and decided to pursue postdoctoral work in a neuroscience-related field in the United States. Recognizing Paolo’s potential, and much to my benefit as it would turn out, Baraldi suggested that he join me in Baltimore to study molecular mechanisms involved in neurodegeneration. During the 1980s, we had performed a series of studies implicating the peripheral tryptophan metabolites (“kynurenines”) quinolinic acid and kynurenic acid in the pathophysiology of Huntington’s Disease (HD) and other neurodegenerative diseases, but we still had only a very rudimentary view of the neurobiology of these compounds when Paolo arrived. Soon after setting foot in the laboratory, Paolo showed that L-kynurenine, rather than tryptophan itself, serves as the primary source of both quinolinate and kynurenate in the brain. In subsequent years, he focused especially on the biology of kynurenate, identifying kynurenine aminotransferase II (KAT II) as its most relevant biosynthetic enzyme in the brain. Using experimental lesions and immunocytochemical methods, he then localized KAT II to astrocytes and suggested that glia-derived kynurenate might play an active role in brain physiology. This idea, which was developed while Paolo was still a postdoctoral fellow, has stood the test of time. Due to the realization that endogenous kynurenate tonically modulates glutamatergic, dopaminergic and cholinergic function, the original concept has, in fact, more recently blossomed into the attractive hypothesis that fluctuations in brain kynurenate control cognitive processes in the mammalian brain. The obvious conjecture—that astrocytic KAT II might be targeted pharmacologically to influence cognitive function—occupied much of Paolo’s time during his final months and would certainly have been a major focus of his career had his life not been tragically cut short.

To readers of this journal, Paolo may be best known for his work on HD. Based on a series of clever experiments in the late 1990s, he concluded that 3-hydroxykynurenine (3-HK), a bioprecursor of quinolinate, may enhance quinolinate-induced neurodegeneration due to the generation of reactive free radicals. In rapid succession, he showed that 3-HK indeed dramatically potentiated the excitotoxic effects of quinolinate in the rat striatum *in vivo* and documented that the brain levels of 3-HK were substantially elevated in several of the newly developed mouse models of HD. These findings, together with his subsequent demonstration that the brain tissue content of both 3-HK and quinolinate is greatly increased in the early stages of the disease, rejuvenated and galvanized the decade-old idea that an excitotoxic mechanism might underlie HD pathology. Moreover, these studies, together with the work of others who had shown that 3-HK and quinolinate are preferentially formed in microglial cells, added an important new twist to the hypothesis, suggesting that neurodegeneration in HD is non-cell autonomous in nature. More specifically, Paolo’s results indicated that the neurotoxic effects of mutant huntingtin, the protein that initiates the pathological cascade, might depend on the activation of microglial kynurenines. As a corollary, it became evident that interventions aimed at preventing or arresting the formation of microglial kynurenines might render the effects of the mutant protein less severe or even innocuous. At the time of his passing, Paolo had designed a number of imaginative genetic and pharmacological approaches to test this attractive hypothesis and its implications for the treatment of HD.

Paolo’s discoveries and the development of his exciting concepts were a direct consequence of his ability to think creatively, his willingness to embark on technically demanding, high-risk projects, an impressive no-nonsense, “can-do” attitude, and—maybe most importantly—an uncanny ability to identify and pursue scientifically important questions. But there was far more to Paolo than talent and professional success. Regular attendees of ISTRY (International Society for Tryptophan Research) meetings will remember him as an energetic, lively and optimistic, and at the same time a kind and generous colleague, to list but a few of the adjectives used to describe Paolo in the many messages sent by mourners from all corners of the world.

Paolo’s approach to life and people brought the best out in everyone, yet his flame shone far too briefly. I will personally miss a wonderful scientific companion, who had a brilliant future ahead of him. There is apparent truth to the old saying that only the good die young. It was a privilege to have him in our lives. He will not be forgotten.

